# Cytotoxicity and infiltration of human NK cells in *in vivo-*like tumor spheroids

**DOI:** 10.1186/s12885-015-1321-y

**Published:** 2015-05-03

**Authors:** Ariane Giannattasio, Sandra Weil, Stephan Kloess, Nariman Ansari, Ernst H K Stelzer, Adelheid Cerwenka, Alexander Steinle, Ulrike Koehl, Joachim Koch

**Affiliations:** 1 NK Cell Biology, Georg-Speyer-Haus, Institute for Tumor Biology and Experimental Therapy, Frankfurt, Germany; 2 Institute for Cellular therapeutics, IFB-Tx, Hannover Medical School, Hannover, Germany; 3 Physical Biology Group, Buchmann Institute for Molecular Life Sciences, Goethe Universität, Frankfurt, Germany; 4 Innate Immunity, German Cancer Research Center, Heidelberg, Germany; 5 Institute for Molecular Medicine, Johann Wolfgang Goethe-University, Frankfurt, Germany

**Keywords:** NK cell, tumor immune escape, tumor infiltration, tumor spheroid, 3D culture, innate immune system, NKG2D, ligand shedding

## Abstract

**Background:**

The complex cellular networks within tumors, the cytokine milieu, and tumor immune escape mechanisms affecting infiltration and anti-tumor activity of immune cells are of great interest to understand tumor formation and to decipher novel access points for cancer therapy. However, cellular *in vitro* assays, which rely on monolayer cultures of mammalian cell lines, neglect the three-dimensional architecture of a tumor, thus limiting their validity for the *in vivo* situation.

**Methods:**

Three-dimensional *in vivo*-like tumor spheroid were established from human cervical carcinoma cell lines as proof of concept to investigate infiltration and cytotoxicity of NK cells in a 96-well plate format, which is applicable for high-throughput screening. Tumor spheroids were monitored for NK cell infiltration and cytotoxicity by flow cytometry. Infiltrated NK cells, could be recovered by magnetic cell separation.

**Results:**

The tumor spheroids were stable over several days with minor alterations in phenotypic appearance. The tumor spheroids expressed high levels of cellular ligands for the natural killer (NK) group 2D receptor (NKG2D), mediating spheroid destruction by primary human NK cells. Interestingly, destruction of a three-dimensional tumor spheroid took much longer when compared to the parental monolayer cultures. Moreover, destruction of tumor spheroids was accompanied by infiltration of a fraction of NK cells, which could be recovered at high purity.

**Conclusion:**

Tumor spheroids represent a versatile *in vivo*-like model system to study cytotoxicity and infiltration of immune cells in high-throughput screening. This system might proof useful for the investigation of the modulatory potential of soluble factors and cells of the tumor microenvironment on immune cell activity as well as profiling of patient-/donor-derived immune cells to personalize cellular immunotherapy.

**Electronic supplementary material:**

The online version of this article (doi:10.1186/s12885-015-1321-y) contains supplementary material, which is available to authorized users.

## Background

Natural killer (NK) cells rapidly recognize and destroy malignantly transformed cells [1-3]. Due to their natural ability to lyse tumor cells without prior sensitization, NK cells hold promise for cancer immunotherapy [4-8]. However, tumor immune escape strategies might compromise NK cell activity, promoting tumor formation and progression of cancer [2]. NK cell cytotoxicity is tightly regulated by a dynamic balance of signals from activating and inhibitory cell surface receptors [1,9]. Among the major activating receptors are the NK group 2 member D receptor (NKG2D) [10-12], the natural cytotoxicity receptors (NCR) [13-15] and DNAM-1 [16]. Human NKG2D recognizes several structurally related ligands (NKG2DLs) on tumor cells from various cytological origin, including the MHC class I chain-related protein A (MICA), MICB, and the UL16-binding proteins (ULBPs) [15,17,18]. Besides their role as tumor antigens, the ectodomains of the NKG2DLs can be shed from the plasma membrane of malignantly transformed cells and subsequently inhibit NKG2D-dependent NK cell cytotoxicity [19,20]. Importantly, tumor infiltration of NK cells coincides with anti-tumor activity and is associated with a better prognosis in several cancer entities such as colorectal cancer, non-small cell lung cancer, and clear cell renal cell carcinoma [21-25]. However, NK cell cytotoxicity might be modulated by cytokines, the cellular crosstalk with tumor-associated cells and tumor immune escape mechanisms within the tumor microenvironment [26].

**Figure 1:**
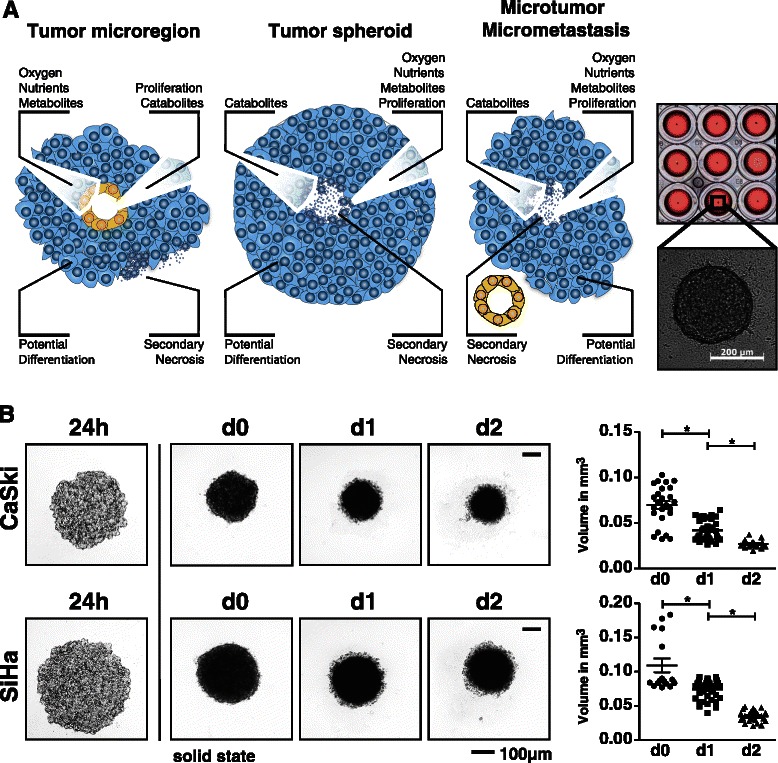
Establishment of tumor spheroids. **(A)** Schematic representation of the microarchitecture of tumor spheroids, avascular tumor microregions and developing micrometastases (based on Friedrich *et al.* [38]) highlighting the pathophysiological similarities and differences. On the right, representative tumor spheroids derived from 2×10^3^ cells are shown in a 96-well plate and by transmission microscopy at 50× magnification. **(B)** Growth kinetics of cervical carcinoma tumor spheroids. 5×10^3^ CaSki or SiHa cells were seeded, and tumor spheroid growth was monitored by phase contrast microscopy at 50× magnification. The solid spheroidal state (day 0 = d0) was used in all further experiments as starting point. Tumor spheroid growth is plotted as the volume of individual spheroids from six independent experiments (n = 6). Data are shown as mean ± SEM. Spheroid volume (in mm^3^) was calculated based on phase contrast image analysis by area determination using Fiji software [35]. The size bar corresponds to 100 μm. A p value < 0.05 is marked as statistically significant (*).

The tumor microenvironment is comprised of malignantly transformed cells, their surrounding stroma (which consists of fibroblasts, endothelial cells, pericytes, and mesenchymal cells), innate immune cells (including macrophages, neutrophils, mast cells, myeloid-derived suppressor cells, dendritic cells, and NK cells), and adaptive immune cells (T and B lymphocytes) [27,28]. Moreover, tumor formation is a highly dynamic process which requires the concerted action of soluble factors and cellular interactions [28]. In order to study cellular networks within a tumor, a model system is required that accounts for this complexity. The majority of published studies is based on monolayer cell culture systems, which lack essential cellular interactions present *in vivo* that are a prerequisite for polarity, differentiation and the establishment of metabolic gradients. Furthermore, 2D cultures do not enable immunosurveillance and infiltration studies. Therefore, the demand to develop three-dimensional cellular model systems is increasing [29,30]. Within the current study, we have established three-dimensional multicellular tumor spheroids, which resemble many features of *in vivo* tumors and allow for systematic investigation of molecular parameters in a defined microenvironment. Tumor spheroid cultures possess a complex network of cell-cell contacts as well as pH, oxygen, metabolic and proliferative gradients reminiscent of the conditions found in poorly vascularized and avascular regions of solid tumors and micrometastases [29,31-34]. Tumor spheroids are formed by association of several thousand cells and are consequently comprised of an outer region of proliferating cells around a body of quiescent cells [31,33]. Moreover, similar to the situation found for benign tumors *in vivo*, tumor spheroids develop a necrotic core once their diameter exceeds the requirements for efficient diffusion of oxygen, nutrients, and metabolites (Figure 1A). Until now, tumor spheroids were mainly used to assess the efficacy and toxicity of drugs in high-throughput screening [29,30].

In the current study, we introduce 3D tumor spheroids as tumor mimic to study NK cell infiltration and immunosurveillance. As a proof of concept, we employed tumor spheroids of two human cervical carcinoma cell lines (SiHa: grade II, human cervix squamous cell carcinoma and CaSki: cervical epidermoid carcinoma). We show that tumor spheroids allow for long-term observation of cell proliferation, NK cell infiltration and NK cell cytotoxicity in the absence and presence of soluble mediators. Importantly, fluorimetric analysis enables the quantification of anti-tumor efficacy. Moreover, magnetic activated cell sorting (MACS) allows for isolation and analysis of immune cells, which have infiltrated into the tumor spheroids.

Based on these data, the current study shows that tumor spheroids represent a novel tool to decipher determinants of tumor immune escape and to study cellular interaction networks in 3D. Therefore, tumor spheroids might proof useful to improve the activity of tumor infiltrating immune cells, which might be important for donor selection of cytotoxic lymphocytes, *status quo* determination of anti-tumor immunoreactivity, and preconditioning of a cancer patient prior to (allogeneic) cellular immunotherapy and thus help to personalize treatment.

## Methods

### Cell culture

Primary NK cells were purified (>95% pure) from buffy coats of healthy donors. Buffy coats were derived from whole-blood donations of healthy volunteer blood donors kindly provided by the German Red Cross Blood Service, Institute for Transfusion Medicine and Immunohematology, Medical School, Goethe-University Frankfurt, Germany. They were used in an anonymized fashion with written donor approval and approval by the Ethics Committee of Goethe University, Frankfurt, permit #329/10. PBMCs were isolated by a density gradient with Biocoll (Biozol, Germany) followed by indirect magnetic immunoselection (Miltenyi Biotec, Germany) and activation in X-Vivo10 medium (Lonza, Switzerland) supplemented with 5% human serum (Life Technologies, USA), 1000 IU/ml IL-2 (Promokine, Germany) and activation beads (Miltenyi Biotec, Germany) for at least 7 days. The cervical carcinoma cell lines CaSki (cervical epidermoid carcinoma) and SiHa (grade II, human cervix squamous cell carcinoma) were kindly provided by A. Cerwenka, DKFZ, Heidelberg, Germany and cultured in DMEM (Life Technologies, USA) basal medium supplemented with 10% FCS (PAA and PAN Biotech, Germany), 1% Penicillin/Streptomycin (Life Technologies, USA) and 2 mM L-Glutamine (Life Technologies, USA).

### Multicellular tumor spheroids

Solid tumor spheroids were generated by seeding 5×10^3^ – 1×10^4^ cells/well in a volume of 150 μl/well of culture medium in 96-well plates coated with 1.5% agarose in basal DMEM medium. Tumor spheroids were used for functional assays upon reaching a solid state approximately 48 h after initial seeding (d0). Growth was monitored by transmission and fluorescence microscopy. For tumor spheroid growth curves, phase contrast pictures of independent solid spheroids were analyzed for each condition from six independent experiments by Fiji software [35]. Tumor spheroid volume was calculated based on area analysis of solid spheroids by Fiji software assuming a perfect sphere.

### Flow cytometry

For the detection of NKG2DLs, single-cell suspensions of monolayer cells or tumor spheroids treated with TrypLE™ Express (Life Technologies, USA) were stained with mouse monoclonal antibodies: mouse anti-human MICA (AMO1[36]), mouse anti-human MICB (MAB1599), mouse anti-human ULBP1 (MAB1380), mouse anti-human ULBP2 (MAB1298) and mouse anti-human ULBP3 (MAB1517, all R&D Systems, USA). Rat anti-mouse IgG_1_-APC (130-095-902, Miltenyi Biotec, Germany) or rat anti-mouse IgG_2a/b_-APC (130-095-880, Miltenyi Biotec, Germany) served as secondary antibodies. As negative control, samples incubated with secondary antibodies only were used. Cells were analyzed with FlowJo software (Tree Star, USA) after measurement on a FACS Canto II instrument equipped with a 96-well plate HTS Sampler. Viability of cells was analyzed by SytoxBlue stain (Life Technologies, USA). 10,000 events of viable cells were analyzed. To calculate x-fold MFI over background, data of individual experiments (n = 3) were normalized by division of the MFI by the MFI of the secondary antibody controls to calculate mean ± SEM.

### ELISA

In order to analyze shedding of NKG2DLs, supernatants of 96 solid tumor spheroids or parental monolayer cultures of counted cell culture flasks were collected at different time points within 72 h (d0, d1, d2) and concentrated 10-fold using Amicon centrifugal filter units (Merckmillipore, Germany). Soluble MICB, ULBP1 and ULBP2 levels were quantified by DuoSet ELISA kits (DY1599, DY1380, DY1298, R&D Systems, USA), following the manufacturer’s instructions. Soluble MICA was analyzed as described [36] with slight modifications. Soluble ULBP3 was detected with a similar protocol [37]. Briefly, ELISA plates were coated with mouse anti-human MICA (AMO1) or goat anti-human ULBP3 antibodies (AF1517, R&D Systems, USA). After saturation with BSA blocking solution (Candor, Germany), the plates were incubated with either samples, recombinant soluble MICA*04 or recombinant ULBP3-Fc (1517-UL-050, R&D Systems, USA) as standards. For detection and quantification of sMICA, the mouse anti-human MICA/B antibody (BAMO3) was used in combination with a goat anti-mouse IgG_2a_ horseradish peroxidase-conjugated antibody (1080–05, Southern Biotechnologies, USA). For sULBP3, the mouse anti-human ULBP3 antibody (CUMO3) was used in combination with a goat anti-mouse IgG_1_ horseradish peroxidase conjugated antibody (1070–05, Southern Biotechnologies, USA). Following visualization with TMB substrate (KPL, Germany), signals were measured in a microtiter plate reader (λ = 450 nm) and analyzed with Prism 5 software (GraphPad, USA). Data were calculated as mean ± SEM per 10.000 cells (per spheroid) of three independent experiments (n = 3), measured in duplicates.

### Functional assays

For monolayer cell cytotoxicity assays CaSki and SiHa monolayer cells were washed, dissociated from cell culture flasks, and the cell suspension was fluorescently labeled with CFSE (Life Technologies, USA). Labeled target cells were incubated for 4 h with primary NK cells at appropriate E:T ratios in NK cell medium without IL-2. For blocking experiments, NK cells were pre-incubated for 30 min with anti-human NKG2D blocking mAb (MAB139, R&D Systems, USA) or anti human TRAIL mAb (EXB-10-316, AXXORA, USA). At the endpoint of the cytotoxicity assay, NK cells were labeled with a mouse anti-human CD45-APC antibody (clone 5B1, Miltenyi Biotec, Germany). Live/dead cell discrimination was achieved by staining with SytoxBlue (Life Technologies, USA). 10,000 events of viable cells were analyzed. The percentage of target cell lysis was calculated by gating on target cells (CFSE^+^) and analysis of SytoxBlue^+^ and SytoxBlue^−^ cells.

Cytotoxicity experiments in tumor spheroids were performed according to the following timeline: i) seeding of tumor cells and formation of tumor spheroids within two days, ii) addition of pre-activated NK cells (d0), iii) monitoring and documentation by light and fluorescence microscopy (d1,d2), and iv) end-point analysis of cytotoxicity by flow cytometry (d1, d2). For cytotoxicity assays, CaSki and SiHa cells were fluorescently labeled with CFSE (Life Technologies, USA) prior to seeding. Upon reaching the solid spheroidal state (d0), NK cells were added at appropriate E:T ratios to the spheroids in NK cell medium without IL-2. Cytotoxicity and spheroid destruction was monitored for 48 h (d1, d2) by brightfield and fluorescence microscopy at 488 nm at 50× magnification. For flow cytometry, single tumor spheroids were dissociated by TrypLE^TM^ Express-treatment. NK cells in these single-cell suspensions were labeled with a mouse anti-human CD45-APC antibody (Miltenyi Biotec, Germany) prior to addition of counting beads (BD, Germany) to calculate cell numbers according to the manufacturer’s instructions. Live/dead cell discrimination was achieved by staining with SytoxBlue (Life Technologies, USA). The percentage of viable cells was calculated by evaluation of the gates for NK cells (CD45^+^/ SytoxBlue^−^), target cells (CFSE^+^/ SytoxBlue^−^), and fluorescent counting beads.

In order to study NK cell infiltration of tumor spheroids, CaSki and SiHa cells were fluorescently labeled with CFSE (Life Technologies, USA) prior to seeding. After reaching the solid spheroidal state, NK cells were added to the spheroids in NK cell medium without IL-2 at an E:T ratio of 3:1 and incubated for 24 h. Viable tumor spheroids were further analyzed by flow cytometry. For flow cytometry, tumor spheroids were harvested with a cut 1000 μl-tip, pooled, and separated from the supernatant containing the surrounding NK cells and residual target cells by centrifugation at 300xg for 10 s. Following two washing steps in a volume of 50 ml PBS/2% FCS, at 300xg for 1 min tumor spheroids were dissociated by TrypLE™ Express-treatment for 5 min. The single cell suspension was washed again and subjected to CD45 MACS-bead isolation according to the manufacturer’s instructions (130-045-801, Miltenyi Biotec, Germany). CD45 MACS beads were chosen for high affinity strong binding to all NK cell subpopulations. Cells from the periphery of the tumor spheroids, and cells in the flow through and elution fractions from MACS, were analyzed by flow cytometry. Tumor spheroids without NK cells and tumor spheroids incubated with NK cells, which had not been submitted to MACS isolation served as controls and were treated with the same procedure. NK cells in the single-cell suspensions were labeled with a mouse anti-human CD45-APC antibody (Miltenyi Biotec, Germany) prior to addition of counting beads (BD, Germany) to calculate cell numbers according to the manufacturer’s instructions. Live/dead cell discrimination was achieved by staining with SytoxBlue (Life Technologies, USA). The percentage of viable cells was calculated by evaluation of the gates for NK cells (CD45^+^/ SytoxBlue^−^), target cells (CFSE^+^/ SytoxBlue^−^), and fluorescent counting beads (Additional file 1: Figure S3). For tumor spheroid arrays, complete samples were analyzed with cellular event counts between 5,000 and 30,000 cells were analyzed with FlowJo software (Tree Star, USA) after measurement on a FACS Canto II instrument equipped with a 96-well plate HTS Sampler.

### Statistical analysis

Statistical analyses were performed with the use of Prism 5 software (GraphPad, USA). Statistical significance of the differences between tumor spheroid volume were calculated by the U Mann–Whitney test. A p value of < 0.05 was considered statistically significant. Data are shown as mean ± SEM as indicated.

## Results

### Generation of tumor spheroid arrays

In order to investigate NK cell cytotoxicity against cervical carcinoma, the cervical carcinoma cell lines CaSki and SiHa were tested for their ability to form solid tumor spheroids (Figure 1B). Tumor spheroid formation was induced by seeding CaSki or SiHa cells into agarose-coated 96-well plates. Spheroid formation was a bi-phasic process of cell aggregation (24 h post seeding, Figure 1B) and spheroid maturation due to cellular rearrangement (48 h post seeding). These mature tumor spheroids (d0) were used as starting point for further experiments. Notably, during the following two days (d1, d2) cellular reorganization continued in the tumor spheroids resulting in moderate compaction and associated significant volume reduction, which did not affect further experiments (Figure 1B). Moreover, since the tumor spheroids remained below a critical size of 500 – 600 μm [38], the tumor spheroids remained solid without signs of central necrosis within five days. The average volume of CaSki spheroids on d0, d1, and d2 was 0.070 ± 0.004 mm^3^, 0.042 ± 0.002 mm^3^, and 0.027 ± 0.001 mm^3^, respectively. The average volume of SiHa spheroids on d0, d1, and d2 was 0.109 ± 0.010 mm^3^, 0.074 ± 0.002 mm^3^, and 0.034 ± 0.001 mm^3^, respectively. Cell viability of >70% after 4 days of culture post seeding (d2) was verified by flow cytometry and live/dead cell analysis by means of SytoxBlue staining. In conclusion, these data demonstrate that individual tumor spheroids grown in parallel show little alterations in size and growth kinetics and are thus perfectly suited for further experiments.

### Tumor spheroids express cellular ligands of NKG2D

**Figure 2:**
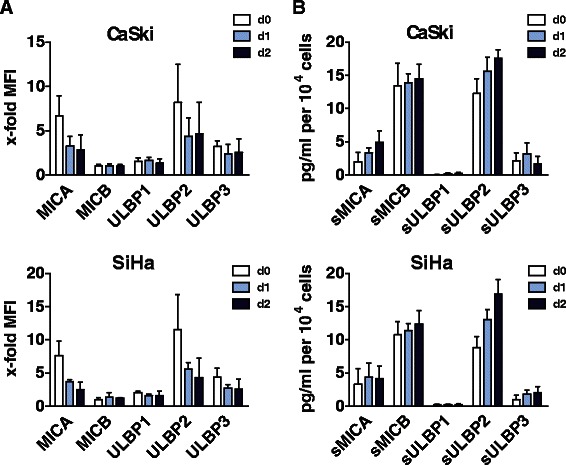
Expression and shedding of ligands of the activating NK cell receptor NKG2D in tumor spheroids of cervical carcinoma. Tumor spheroids grown from CaSki and SiHa cells were analyzed for the expression and release of soluble NKG2DLs. Tumor spheroid formation was induced by seeding 10^4^ cells into 1.5 % agarose-coated 96-wells. **(A)** Expression of NKG2DLs in tumor spheroids collected on day 0 (d0), day 1 (d1) and day 2 (d2) analyzed by flow cytometry. Data are presented as mean ± SEM of three independent experiments (n = 3), measured in duplicates. **(B)** Kinetics of soluble NKG2DL release. Supernatants of tumor spheroids were collected on three consecutive days (d0 – d2) and concentrated 10-fold. Shedding of sMICA, sMICB, sULBP1, sULBP2 and sULBP3 was quantified by ELISA. Data are shown as mean ± SEM in pg/ml per 10.000 cells of three independent experiments (n = 3), measured in duplicates.

A prerequisite for NK cell immunosurveillance of malignantly transformed cells is the presence of tumor antigens, which induce downstream signaling of activating NK cell receptors and related cytotoxicity. In this context, tumor spheroid culture may influence pathophysiological stress levels and cell-cell contacts [29]. Therefore, we examined the plasma membrane expression of the stress-induced tumor antigens MICA, MICB, ULBP1, ULBP2 and ULBP3 (collectively termed NKG2DLs) on the tumor spheroids for comparison with the expression levels found in the corresponding parental monolayer cultures (Figure 2A, Additional file 2: Figure S1, representative histograms Additional file 2: Figure S1B). As a result, CaSki and SiHa cells derived from monolayer cultures displayed a robust expression of MICA, ULBP2 and ULBP3 but showed only weak expression of MICB and ULBP1. Interestingly, CaSki and SiHa cells derived from tumor spheroid culture showed the same signature of NKG2DL expression, however, the overall level of NKG2DLs was decreased in tumor spheroids as demonstrated by flow cytometry (Figure 2A, Additional file 2: Figure S1A, B). Moreover, these results are supported by immunohistochemistry investigating NKG2DLs’ expression on cryosections of tumor spheroids (data not shown). Notably, the plasma membrane levels of NKG2DLs decreased over 3 days of tumor spheroid culture as a result of ligand shedding from the plasma membrane (see below). By contrast, the expression level of HLA-A, −B, −C and -E was preserved at both culture conditions (data not shown).

### Tumor spheroids shed cellular ligands of NKG2D

Many cancer cells are known to release soluble NKG2D ligands (soluble NKG2DLs) as part of a tumor immune escape strategy [19]. Therefore, supernatants of solid tumor spheroids were probed for the levels of soluble MICA (sMICA), MICB (sMICB), ULBP1 (sULBP1), ULBP2 (sULBP2) and ULBP3 (sULBP3) over three days (d0, d1, d2) by ELISA. Due to continuous shedding, soluble NKG2DLs accumulated in the supernatant of CaSki and SiHa spheroids reaching saturation after three days of culture (Figure 2B). The predominant ligands shed into the supernatant of tumor spheroids grown from both cell lines within two days were sMICA (CaSki: 4.9 ± 1.7 pg/ml per 10,000 cells; SiHa: 4.2 ± 1.8 pg/ml per 10,000 cells), sMICB (CaSki: 14.5 ± 2.2 pg/ml per 10,000 cells; SiHa: 12.4 ± 2.0 pg/ml per 10,000 cells), and sULBP2 (CaSki: 17.6 ± 2.3 pg/ml per10,000 cells; SiHa: 16.9 ± 2.2 pg/ml per 10,000 cells), whereas sULBP3 was low and sULBP1 levels were below the detection limit of the assay (<2 pg/ml). Notably, the kinetics of soluble NKG2DL release into the supernatant parallels the loss of MICA, MICB, ULBP2 and ULBP3 from the cell surface (Figure 2A). This pattern of sNKG2DL release is in agreement with parental monolayer cultures (Additional file 2: Figure S1A). Since the generated tumor spheroids display a robust NKG2DL signature, we conclude, that the cervical carcinoma spheroids represent a suitable model system for further NK cell studies.

### Primary NK cells infiltrate and destroy tumor spheroids

**Figure 3:**
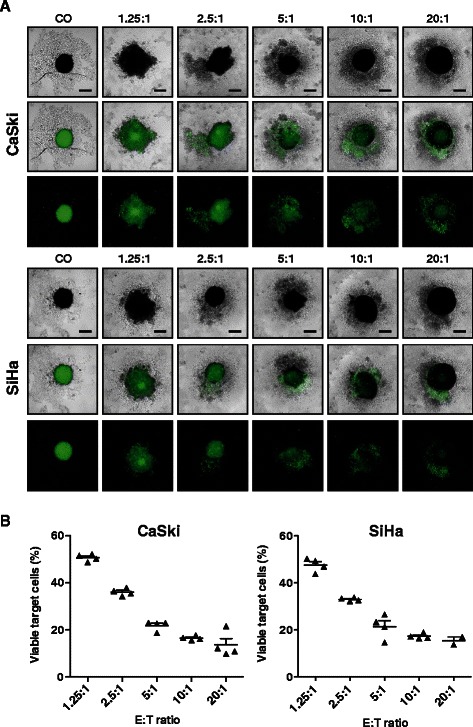
NK cell cytotoxicity against tumor spheroids of cervical carcinoma. Tumor spheroids grown from CFSE-labeled CaSki or SiHa cells (d0) were co-cultured with primary human NK cells at different effector-to-target (E:T) ratio for 48 h. Tumor spheroids without NK cells served as controls (CO). **(A)** Tumor spheroid destruction was monitored by transmission and fluorescence microscopy at 100× magnification. Size bars correspond to 200 μm. **(B)** Flow cytometric analysis of residual viable target cells. Individual tumor spheroids subjected to cytotoxicity assays with NK cells were disintegrated, and NK cells were stained for CD45. The percentage of residual viable cells was calculated by evaluation of the gates for NK cells (CD45^+^/SytoxBlue^−^) and target cells (CFSE^+^/SytoxBlue^−^). Dot blots show the percentage of viable target cells in individual tumor spheroids. Data are shown as mean ± SEM of a representative experiment.

The main focus of this study was to establish a 3D tumor model to study infiltration and cytotoxicity of NK cells. The following issues were important: 1) easy to handle set up, 2) implementation of commonly available materials and analysis methods, and 3) applicability for high-throughput screening. In order to monitor tumor spheroid integrity and destruction, CaSki and SiHa cells were labeled with CFSE, a common cell-tracer, which allows for simultaneous long-term observation of cell proliferation and cell migration. CFSE fluorescence was stable for 6 days in CaSki and SiHa spheroids. To study NK cell infiltration into tumor spheroids and NK cell cytotoxicity, primary human NK cells (*ex-vivo* IL-2 pre-activated and expanded) were added to solid tumor spheroids grown from CaSki and SiHa cells at different effector-to-target (E:T) ratios and co-cultured for several days. Tumor spheroid integrity was monitored by light and fluorescence microscopy (Figure 3). A co-culture period of 48 h was identified as optimal end-point to quantify NK cell cytotoxicity. During tumor spheroid destruction, NK cells accumulated and proliferated in the periphery of the tumor spheroid (Additional file 2: Figure S1C). The degree of NK cell cytotoxicity was monitored based on the number of target cells with persistent CFSE fluorescence. These viable target cells have, by contrast to lysed target cells, not released intracellular CFSE due to loss of cellular integrity (Figure 3). Fluorescence microscopy revealed that solid tumor spheroids grown in the absence of NK cells were homogenously stained whereas the overall number of viable and thus fluorescent target cells dramatically decreased with increasing E:T ratios as a result of NK cell cytotoxicity (Figure 3A). To quantify NK cell cytotoxicity, we developed a flow cytometry protocol for the analysis of single tumor spheroids. Briefly, individual tumor spheroids and NK cells were transferred well-by-well into an agarose-free 96-well plate. Following sedimentation of the tumor spheroids by gentle centrifugation, supernatants were aspirated, and solid tumor spheroids were disintegrated by enzymatic digest. Cell suspensions derived from individual tumor spheroids were washed, and the NK cells were labeled with CD45-specific antibodies. In the next step, the dead-cell marker SytoxBlue and fluorescent counting beads were added to the 96-wells to quantify residual viable tumor cells. Target cell lysis was quantified based on the number of CFSE^+^/ CD45^−^/ SytoxBlue^−^ target cells. The flow cytometry analysis of single spheroids showed an E:T dependent decrease of target cells (Figure 3B), which correlated with the number of CFSE positive cells as determined by fluorescence microscopy (Figure 3A). Notably, samples were analyzed by applying a HTS-sampler for sample loading in 96-well format. Thus, NK cell cytotoxicity of individual spheroids can be analyzed by microscopy and flow cytometry in high-throughput screening mode.

### Infiltrated NK cells can be recovered from tumor spheroids

**Figure 4:**
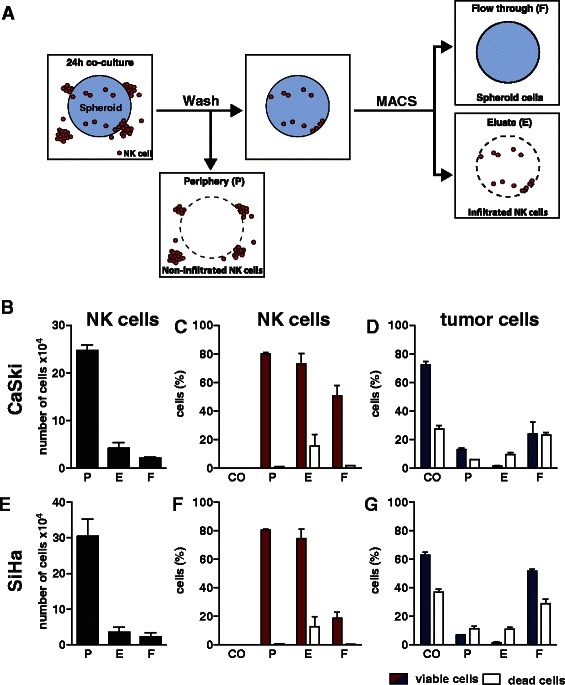
Isolation and enrichment of infiltrated NK cells from tumor spheroids of cervical carcinoma. **(A)** Work flow of the cell fractionation. 96 tumor spheroids grown from CFSE-labeled CaSki or SiHa cells (d0) were co-cultured with primary human NK cells at an effector-to-target (E:T) ratio of 3:1 for 24 h. Tumor spheroids were collected, pooled, and non-infiltrated NK cells and residual target cells in the periphery of the tumor spheroids (P fraction) were removed by washing. Solid spheroids containing infiltrated NK cells were disintegrated and subjected to CD45 MACS-bead isolation leading to a flow through fraction (F fraction) containing spheroid cells and an elution fraction (E fraction) containing the CD45^+^ NK cells. Cells from all fractions were quantified by flow cytometry after SytoxBlue staining for discrimination of viable and dead cells. Tumor spheroids incubated in the absence of NK cells served as cytotoxicity control (CO). **(B/E)** Quantification of infiltrated NK cells isolated from tumor spheroids derived from CaSki **(B)** and SiHa **(E)** cells. Data are shown as total number of CD45^+^ cells per sample. **(C/D/F/G)** Analysis of cell viability. Proportions of viable and dead NK cells **(C/F)** and target cells **(D/G)** within the fractions are shown. Viable cells are shown as solid bars, dead cells as open bars. Data are shown as mean ± SEM of three individual experiments (n = 3).

Infiltration of NK cells is a prerequisite for tumor spheroid destruction. However, in transmission microscopy, we frequently observed that most NK cells were localized in proliferation cones in the periphery of the tumor spheroids, whereas only a small fraction of the applied NK cells infiltrated into the tumor spheroid (Additional file 3: Figure S2 and Video S1). To further characterize these subpopulations of infiltrated and non-infiltrated NK cells, we established a MACS-based protocol to isolate and quantify infiltrated NK cells (Figure 4A). Briefly, solid CaSki and SiHa tumor spheroids were co-incubated with human primary NK cells at a low E:T ratio (3:1) for 24 h to reduce destruction of the tumor spheroids and to allow for infiltration studies. Solid tumor spheroids grown in the absence of NK cells served as negative control. Peripheral NK cells were separated from the residual solid tumor spheroids by centrifugation. Isolated tumor spheroids were disintegrated by enzymatic digestion, and NK cells were recovered by MACS isolation after specific labeling with CD45-magnetobeads. The three resulting cell fractions are i) peripheral non-infiltrating NK cells in the supernatant after centrifugation (P), ii) tumor cells in the flow through of the MACS column (F), and iii) infiltrated NK cells recovered after elution from the MACS column (E) (Figure 4A). Discrimination of tumor cells and NK cells was achieved by means of CFSE fluorescence and specific labeling of the NK cells with CD45-specific antibodies. SytoxBlue and fluorescent counting beads were added to the samples to quantify infiltrated NK cells and residual viable cells.

Flow cytometric analysis of the individual cell fractions showed that the majority of NK cells did not infiltrate and were detected in the periphery. Most importantly, for further analysis, we have recovered 24.8 ± 1.2 ×10^4^ and 30.5 ± 4.8 ×10^4^ NK cells from the periphery and 4.2 ± 1.2 ×10^4^ and 3.5 ± 1.3×10^4^ infiltrated NK cells from the elution fractions of CaSki and SiHa spheroids, respectively (Figure 4B and E). These cell numbers correspond to 20.5% and 16.2% of total NK cells infiltrated into CaSki and SiHa tumor spheroids (E and F fraction, Figure 4B and E). The purity of recovered infiltrated NK cells (E fraction) was very high with 96.0% and 97.1% viable NK cells for CaSki and SiHa tumor spheroids (E fraction contained 1.7 ± 0.3% viable and 9.7 ± 1.2% dead tumor cells for CaSki and 1.7 ± 0.7% viable and 11.0 ± 1.5% dead tumor cells for SiHa tumor spheroids, Figure 4D and G). Interestingly, more than 98.0% of the peripheral NK cells were viable (P fraction: 80.0 ± 1.0% viable and 1.0 ± 0.0% dead NK cells for CaSki, 80.5 ± 0.5% viable and 1.0 ± 0.0% dead NK cells for SiHa tumor spheroids), whereas the viability of the infiltrated NK cells was slightly lower with 82.4% and 85.4% viable NK cells derived from CaSki and SiHa tumor spheroids (E fraction: 73.0 ± 7.4% viable and 15.7 ± 7.9% dead NK cells for CaSki, 74.3 ± 6.9% viable and 12.7 ± 7.1% dead NK cells for SiHa tumor spheroids, Figure 4C and F). Moreover, analysis of tumor cell viability by SytoxBlue staining demonstrated that residual viable target cells were detected in the flow through (F fraction: 24.0 ± 8.5% viable and 23.0 ± 1.9% dead tumor cells for CaSki, 51.7 ± 1.4% viable and 29.0 ± 3.2% dead tumor cells for SiHa tumor spheroids) and also in the periphery (P fraction: 13.0 ± 1.0% viable and 6.0 ± 0.0% dead tumor cells for CaSki, 7.0 ± 0.0% viable and 11.5 ± 1.5% dead tumor cells for SiHa tumor spheroids, Figure 4D and G). Consequently tumor cells were efficiently killed by primary NK cells after 24 h co-incubation, showing 59.9% and 45.8% dead cells for CaSki and SiHa tumor spheroids (F fraction), respectively. For reference, tumor spheroids, which were grown in the absence of NK cells showed only 26.8% and 36.3% dead tumor cells for CaSki and SiHa tumor spheroids, respectively. Notably, tumor spheroid destruction by NK cells was incomplete due to the low E:T ratio (3:1) used in these experiments focused on NK cell infiltration. NK cell killing of CaSki and SiHa cells showed a strong dependency on the activating receptor NKG2D as demonstrated in monolayer cytotoxicity assays in the absence and presence of NKG2D blocking antibodies (Additional file 2: Figure S1A). In the presence of NKG2D blocking antibodies killing of CaSki and SiHa cells was reduced by 55.7% and 85.1%, respectively. To address NKG2D-dependent infiltration and cytotoxicity in the tumor spheroid system, we used anti-NKG2D antibodies in blocking experiments. In this context, the number of infiltrated NK cells was reduced by 18.4% for CaSki and by 3% for SiHa spheroids whereas NK cell viability was not affected (data not shown). The molecular details of this phenomenon are currently not understood. However, a recent study by Raulet and colleagues [39] showed that shed ligands of NKG2D might lead to activation of NK cell cytotoxicity. Therefore, the tumor spheroid system presented in the current study might be helpful to characterize properties of NKG2DLs which might only be seen in an *in vivo*-like situation.

## Discussion

Tumor spheroids have been widely applied in investigative drug screens for chemo- and radiosensitivity [29,40] of solid cancers of epithelial origin, and their metabolic and proliferative profiles have been well determined. Moreover, tumor spheroids generated from CaSki and SiHa cells were used to investigate gene expression profiles, calcium binding, signaling, drug metabolism and carcinogenesis [41-47]. However, only few reports exist employing tumor spheroids to study immunosurveillance of tumor cells. In this context, previous studies showed NK cell resistance of Ewing’s sarcoma cells that were cultured in a 3D culture model [48] and PBMCs’ and γδ T cells’ activity in tumor spheroids [49,50]. In the clinics, there is accumulating evidence that immune cell invasion into solid tumors of different entities is an important factor for survival of patients [21,51-53] and also for the success of cellular therapies [4]. Although NK cells hold promise for cancer therapy, NK cell infiltration into tumors and their phenotype and function within the tumor has not yet been extensively investigated, partly due to a lack of appropriate model systems [22-25,54-57]. Since, tumor spheroid cultures possess a complex network of cell-cell contacts as well as pH, oxygen, metabolic and proliferative gradients [29,31-33], the model can also help to solve immunological questions of solid tumors, which cannot be addressed in monolayer cultures e.g. the impact of necrotic, hypoxic or hyperproliferative areas in tumor sites and their influence on NK cell functions.

Here we present *in vivo*-like 3D tumor spheroids as a model system for the long-term analysis of tumor immunosurveillance and immune cell infiltration. The present study is the first report investigating NK cell immunosurveillance at *in vivo*-like conditions. This novel application of tumor spheroids enables the characterization of infiltrating cells using commercially available reagents and commonly used cell biological methods. Furthermore, the used 96-well plate format can easily be adapted to high-throughput screening. Within the current study we show an E:T ratio-dependent infiltration and destruction of tumor spheroids by primary NK cells. Interestingly, destruction of a three-dimensional tumor spheroid took much longer when compared to the parental monolayer cultures, highlighting their validity for the *in vivo* situation. Moreover, destruction of tumor spheroids was accompanied by infiltration of a fraction of NK cells, which could be recovered at high purity.

CaSki and SiHa spheroids and parental monolayer cells expressed ligands for the activating NK cell receptor NKG2D. However, sMICA, sMICB, and sULBP2 were continuously shed from the tumor spheroids reaching saturation after approximately 48 h. This rapid re-production and accumulation of soluble NKG2DLs in the supernatant, highlights their role in tumor immune escape [6,58]. Based on CaSki and SiHa tumor spheroids with an average spheroid volume of 0.1 mm^3^, a single CaSki spheroid of 10,000 cells releases 38.97 pg soluble NKG2DLs and a single SiHa spheroid 35.78 pg within 5 days. Typical concentrations of soluble NKG2DLs in cancer patients are 0.4-10 ng/ml [6]. Taking into account the blood volumes of infants (85 ml/kg) and adults (65 ml/kg) and the serum half-life of soluble NKG2D ligands of 6 days [59], the tumor mass required to account for the plasma levels of soluble NKG2DLs in patients can be estimated to tumor spheres with a diameter of 5.9 - 17.8 mm and 10.9 - 32.7 mm, respectively. Notably, the required tumor mass can be scattered throughout the body (e.g. micrometastases) as tumor foci of a size below the diagnostic detection limit. Furthermore, the calculated tumor mass might even be smaller, since NKG2DLs could be released by non-tumor cells under stress conditions during cancer therapy. The panel of ligands expressed, the route of ligand release and the local concentrations of soluble components in a given microenvironment might differ from patient to patient [60].

Since NK cell infiltration is the prerequisite for NK cell function, we focus on NK cell infiltration into tumor spheroids. We show for the first time anti-tumor efficacy of NK cells and efficient tumor spheroid infiltration of a small number of primary activated NK cells into tumor spheroids grown from cervical carcinoma. We assume that these NK cell subpopulations could be of differential phenotype in agreement with Horowitz *et al.* who showed the existence of more than 6,000-30,000 phenotypically distinct NK cell subsets in the blood of a single human [61,62] and with Bhat *et al.* who showed that NK cells were not equally active in forming contacts and killing targets but few ‘hyperactive’ NK cells were observed in cytotoxicity assays [63]. Different factors of immunoediting and immunoevasion could also affect the anti-tumor activity of NK cells [26] and the cellular crosstalk with other cells within the tumor [64,65]. Further characterization of these isolated subpopulations will be instrumental to decipher which factors determine infiltration into solid tumors and will help to improve cellular therapies of solid cancers.

## Conclusions

Taken together, tumor spheroids are a more appropriate model system for *in vivo* tumors than conventional monolayer cultures and allow for stable, reproducible, long-term co-culture with NK cells and also with other immune cells. The presented methods offer the possibility to use the tumor spheroid model system in different ways. Single tumor spheroids can be used i) to monitor tumor growth, ii) to investigate immunosurveillance and associated immune escape by soluble mediators, iii) to investigate tumor spheroid infiltration and spheroid destruction, and iv) to analyze cellular interaction networks by fluorescence microscopy and flow cytometry. Most importantly, infiltrated NK cell subpopulations can be quantified and characterized after isolation. In addition, spheroids can be sectioned and stained for NK cell infiltration as well as for apoptosis by immunohistochemistry. The tumor spheroid model system and the described methods can be adapted to many cell lines of different solid cancer entities of epithelial origin (a comprehensive list of suitable cell lines can be found at [38,66]) as well as to different cytotoxic lymphocyte populations. Furthermore, stromal cells and other cell types that might affect the tumor micromilieu can be included to introduce further complexity of the tumor microenvironment.

Therefore, tumor spheroids represent a versatile *in vivo*-like model system to study cytotoxicity and infiltration of immune cells and might proof useful for the investigation of the modulatory potential of soluble factors and cells of the tumor microenvironment on immune cell activity as well as profiling of patient-/donor-derived immune cells to personalize cellular immunotherapy.

## Additional files

Additional file 1: Figure S3. Gating strategy for flow cytometry assays. In order to study NK cell infiltration of tumor spheroids, CaSki and SiHa cells were fluorescently labeled with CFSE prior to seeding. After reaching the solid spheroidal state, NK cells were added to the spheroids in NK cell medium without IL-2 at a low E:T ratio of 3:1 and incubated for 24 h. Viable tumor spheroids were further analyzed by flow cytometry. NK cells in the single-cell suspensions were labeled with a mouse anti-human CD45-APC antibody prior to addition of counting beads to calculate cell numbers according to the manufacturer’s instructions. Live/dead cell discrimination was achieved by staining with SytoxBlue. The event counts of viable cells were calculated by evaluation of the gates for NK cells (CD45^+^) target cells (CFSE^+^), and fluorescent counting beads. Cells were analyzed on a FACS Canto II instrument with FlowJo software.
Additional file 2: Figure S1. Expression and shedding of ligands for the activating NK cell receptor NKG2D in monolayer culture and NKG2D-dependent cytotoxicity of NK cells against cervical carcinoma cells. **(A)** NKG2DL expression levels in cervical carcinoma cell lines. CaSki and SiHa cells grown as monolayer culture were stained for expression of NKG2DLs and analyzed on day 0 (d0), day 1 (d1) and day 2 (d2) by flow cytometry. Background-corrected MFI of three independent experiments (n = 3) measured in duplicates are shown as mean ± SEM. Kinetics of soluble NKG2DL release. Supernatants of monolayer cultures were collected on three consecutive days (d0 – d2) and concentrated 10-fold. Shedding of sMICA, sMICB, sULBP1, sULBP2 and sULBP3 was quantified by ELISA. Data are shown as mean ± SEM in pg/ml per 10^4^ cells of three independent experiments (n = 3), measured in duplicates. NK cell cytotoxicity assays. CaSki or SiHa cells were co-cultured with primary human NK cells for 4 h in different E:T ratios and analyzed by flow cytometry. Target cells were labeled with CFSE prior to seeding, and NK cells were labeled with anti-CD45 antibodies. The degree of target cell lysis was calculated by gating on CD45^−^/ CFSE^+^ cells and analysis of dead SytoxBlue^+^ or viable SytoxBlue^−^ cells. Specific blocking antibodies were used to determine NKG2D- and TRAIL-dependency of NK cell cytotoxicity. Data are shown as mean ± SEM of duplicates of a representative experiment. **(B)** Representative flow cytometry histograms of CaSki and SiHa cells grown as monolayer culture or as tumor spheroids stained for expression of NKG2DLs are shown on day 2. **(C)** A representative tumor spheroid is shown after 1 h co-incubation with primary NK cells at an effector-to-target ratio of 5:1 by phase contrast microscopy at 50× magnification. The size bar corresponds to 500 μm.
Additional file 3: Figure S2 and Video S1. Video sequence of tumor spheroid infiltration by NK cell. Tumor spheroids were co-cultured in a low effector-to-target (E:T) ratio of 2.5:1 with primary NK cells to favor infiltration events. In order to visualize the tumor spheroids, they were labeled with the photo-labile CFSE-dye. Therefore, disappearance of green fluorescence within 48 h of observation results from photo-bleaching of the CFSE-dye, rather than destruction of tumor spheroid cells. Moreover, photo-bleaching allows for visualization of infiltrated NK cells (labeled with photo-stabile Hoechst-red-dye) with improved contrast. Live cell imaging was performed on a Carl Zeiss Cell Observer Spinning Disk microscope. Pictures were taken with a 10× NA 0.3 objective lens every 30 min over 48 h (representative video sequence). Pictures derived from the video sequence (same focal plane) at different time points are shown. The dashed line indicates the borders of the tumor spheroid from time point 0 (Figure S2).
