# Supplementary figures and images for: Cytotoxicity and infiltration of human NK cells in in vivo-like tumor spheroids
Source: BMC Cancer. 2015 May 3;15:351. doi: 10.1186/s12885-015-1321-y (PMC4422268; doi:10.1186/s12885-015-1321-y)

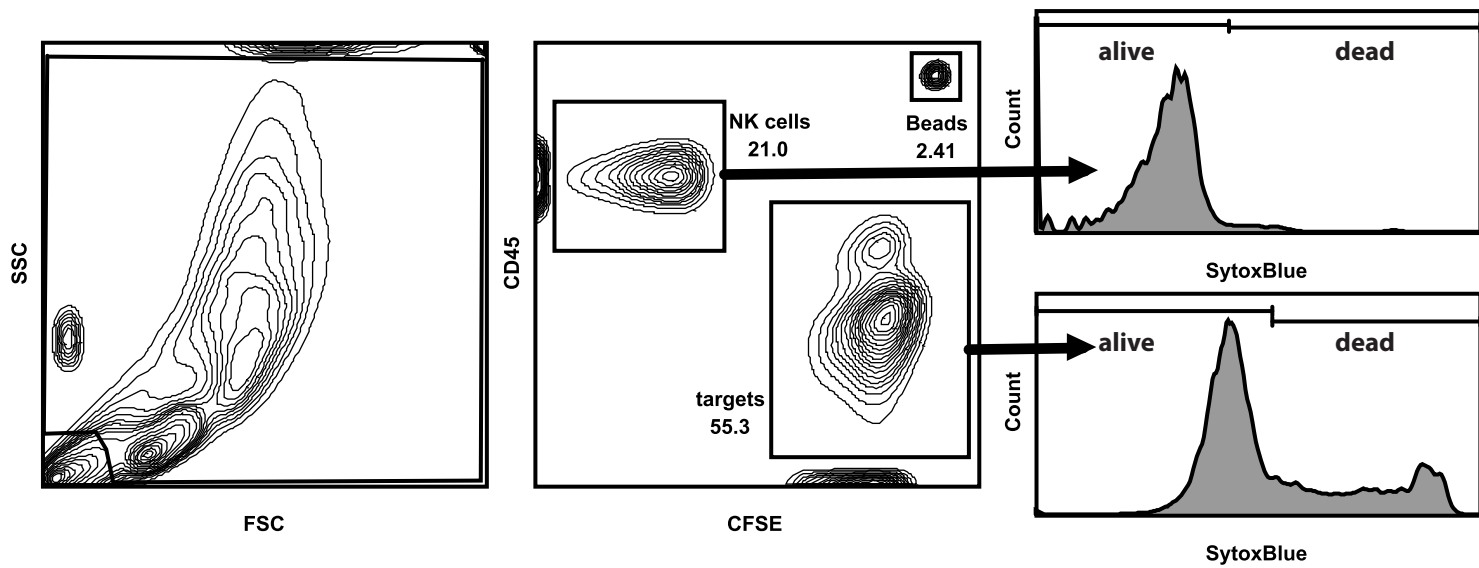

Supplement: Additional file 1: Figure S3. — Gating strategy for flow cytometry assays. In order to study NK cell infiltration of tumor spheroids, CaSki and SiHa cells were fluorescently labeled with CFSE prior to seeding. After reaching the solid spheroidal state, NK cells were added to the spheroids in NK cell medium without IL-2 at a low E:T ratio of 3:1 and incubated for 24 h. Viable tumor spheroids were further analyzed by flow cytometry. NK cells in the single-cell suspensions were labeled with a mouse anti-human CD45-APC antibody prior to addition of counting beads to calculate cell numbers according to the manufacturer’s instructions. Live/dead cell discrimination was achieved by staining with SytoxBlue. The event counts of viable cells were calculated by evaluation of the gates for NK cells (CD45+) target cells (CFSE+), and fluorescent counting beads. Cells were analyzed on a FACS Canto II instrument with FlowJo software. [file 12885_2015_1321_MOESM1_ESM.pdf]

**A**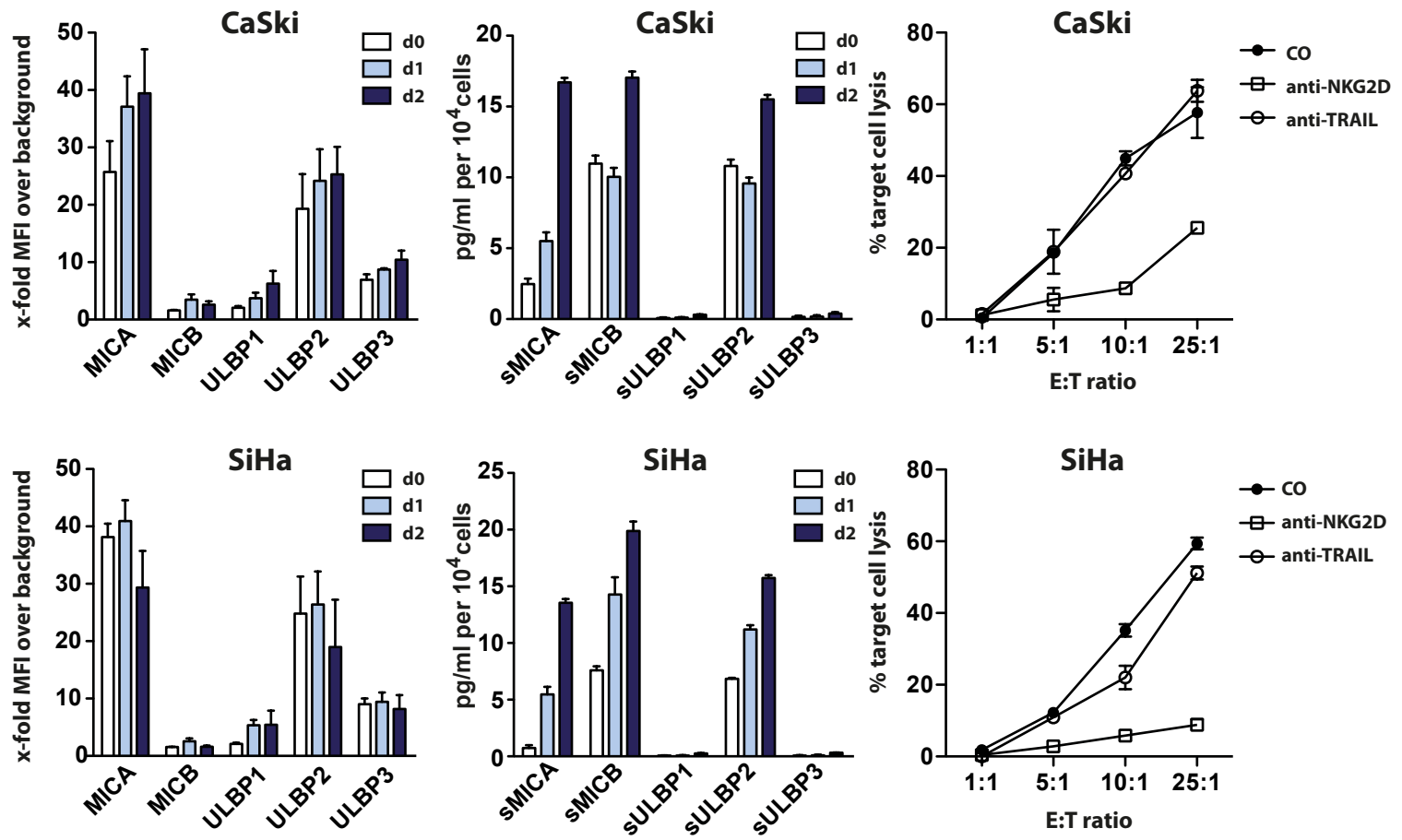**B Monolayer**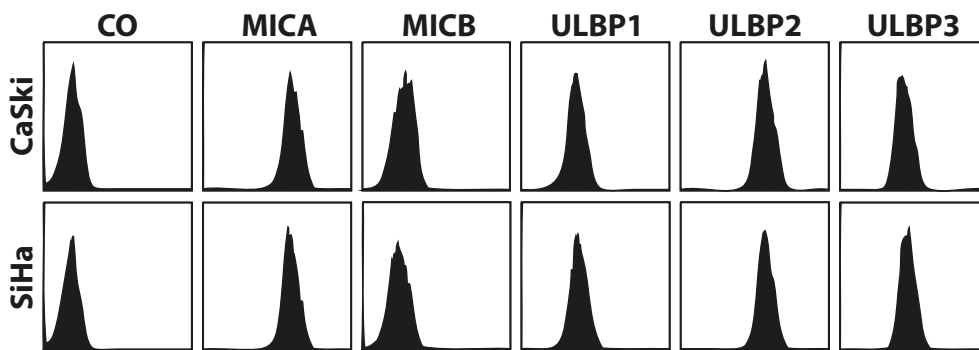**Spheroids**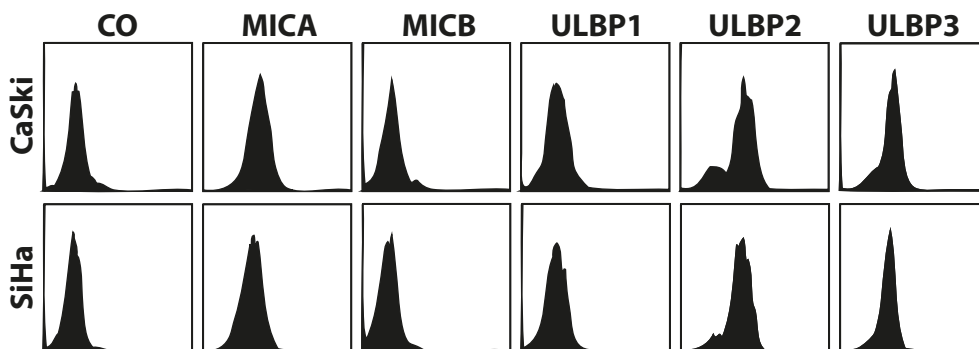**C**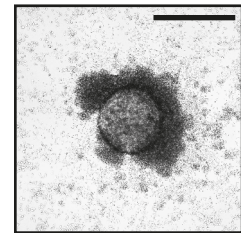

Supplement: Additional file 2: Figure S1. — Expression and shedding of ligands for the activating NK cell receptor NKG2D in monolayer culture and NKG2D-dependent cytotoxicity of NK cells against cervical carcinoma cells. (A) NKG2DL expression levels in cervical carcinoma cell lines. CaSki and SiHa cells grown as monolayer culture were stained for expression of NKG2DLs and analyzed on day 0 (d0), day 1 (d1) and day 2 (d2) by flow cytometry. Background-corrected MFI of three independent experiments (n = 3) measured in duplicates are shown as mean ± SEM. Kinetics of soluble NKG2DL release. Supernatants of monolayer cultures were collected on three consecutive days (d0 – d2) and concentrated 10-fold. Shedding of sMICA, sMICB, sULBP1, sULBP2 and sULBP3 was quantified by ELISA. Data are shown as mean ± SEM in pg/ml per 104 cells of three independent experiments (n = 3), measured in duplicates. NK cell cytotoxicity assays. CaSki or SiHa cells were co-cultured with primary human NK cells for 4 h in different E:T ratios and analyzed by flow cytometry. Target cells were labeled with CFSE prior to seeding, and NK cells were labeled with anti-CD45 antibodies. The degree of target cell lysis was calculated by gating on CD45−/ CFSE+ cells and analysis of dead SytoxBlue+ or viable SytoxBlue− cells. Specific blocking antibodies were used to determine NKG2D- and TRAIL-dependency of NK cell cytotoxicity. Data are shown as mean ± SEM of duplicates of a representative experiment. (B) Representative flow cytometry histograms of CaSki and SiHa cells grown as monolayer culture or as tumor spheroids stained for expression of NKG2DLs are shown on day 2. (C) A representative tumor spheroid is shown after 1 h co-incubation with primary NK cells at an effector-to-target ratio of 5:1 by phase contrast microscopy at 50× magnification. The size bar corresponds to 500 μm. [file 12885_2015_1321_MOESM2_ESM.pdf]

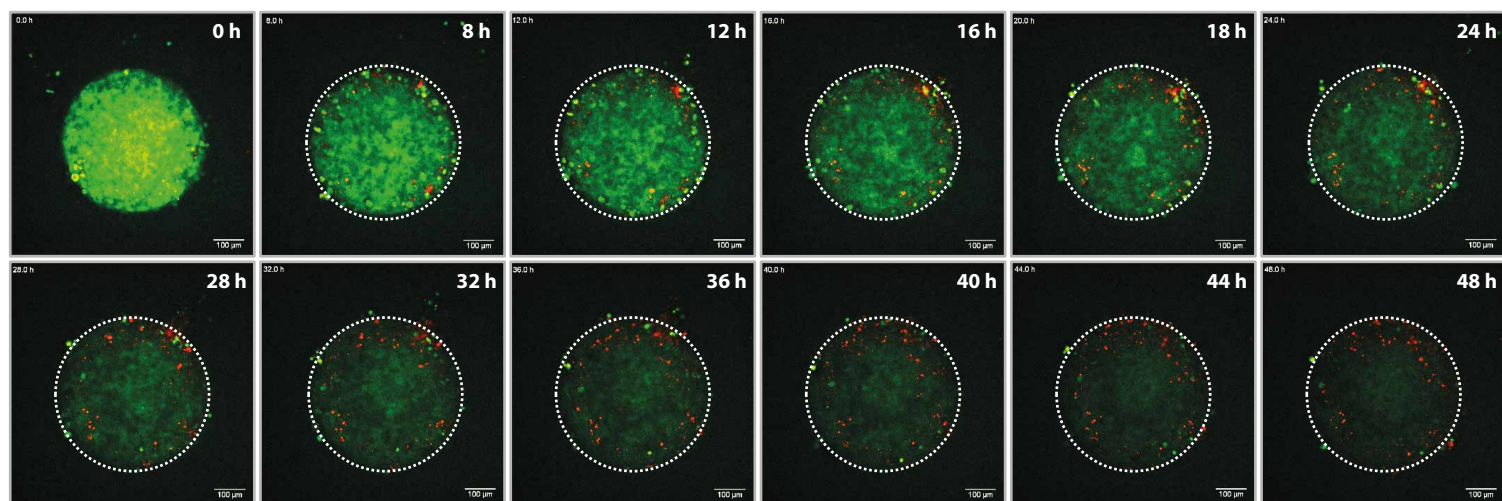

Supplement: Additional file 3: Figure S2 and Video S1. — Video sequence of tumor spheroid infiltration by NK cell. Tumor spheroids were co-cultured in a low effector-to-target (E:T) ratio of 2.5:1 with primary NK cells to favor infiltration events. In order to visualize the tumor spheroids, they were labeled with the photo-labile CFSE-dye. Therefore, disappearance of green fluorescence within 48 h of observation results from photo-bleaching of the CFSE-dye, rather than destruction of tumor spheroid cells. Moreover, photo-bleaching allows for visualization of infiltrated NK cells (labeled with photo-stabile Hoechst-red-dye) with improved contrast. Live cell imaging was performed on a Carl Zeiss Cell Observer Spinning Disk microscope. Pictures were taken with a 10× NA 0.3 objective lens every 30 min over 48 h (representative video sequence). Pictures derived from the video sequence (same focal plane) at different time points are shown. The dashed line indicates the borders of the tumor spheroid from time point 0 (Figure S2). [file 12885_2015_1321_MOESM3_ESM.zip › additonal file 2/12885_2015_1321_MOESM2_ESM.pdf]
